# Predicting Flossing through the Application of the Multi-Theory Model (MTM) of Health Behavior Change among Minority Adolescents in the United States

**DOI:** 10.3390/ijerph192215106

**Published:** 2022-11-16

**Authors:** Manoj Sharma, Kavita Batra, Ching-Chen Chen, Chia-Liang Dai, Ravi Batra, David P. Cappelli

**Affiliations:** 1 Department of Social and Behavioral Health, School of Public Health, University of Nevada, Las Vegas, NV 89119, USA; 2 Department of Medical Education, Kirk Kerkorian School of Medicine at UNLV, University of Nevada, Las Vegas, NV 89102, USA; 3 Office of Research, Kirk Kerkorian School of Medicine at UNLV, University of Nevada, Las Vegas, NV 89102, USA; 4 Department of Counselor Education, School Psychology, and Human Services, College of Education, University of Nevada, Las Vegas, NV 89154, USA; 5 Department of Teaching and Learning, College of Education, University of Nevada, Las Vegas, NV 89154, USA; 6 Department of Information Technology, Coforge Ltd., Atlanta, GA 30338, USA; 7 School of Dental Medicine, University of Nevada, Las Vegas, NV 89102, USA

**Keywords:** oral health, flossing, adolescents, African American/black, Latinx/Hispanic adolescents, health disparities

## Abstract

Adolescents from minority groups are particularly susceptible to poor oral hygiene behaviors, including lack of daily flossing. This cross-sectional study aimed to conduct an exploratory behavioral research to identify evidence-based (theory-based) approaches to promote flossing behavior among African American/Black and Latinx/Hispanic (minority) adolescents. A 39-item psychometrically valid web-based questionnaire was used to collect responses from a nationwide sample of minority adolescents aged 10–17 years residing in the United States. The data were analyzed using bivariate and multivariate statistical methods. Of 520 minority adolescents (260 African American/Black and 260 Latinx/Hispanic adolescents), the proportion of flossing was nearly equally split in the sample. A significantly higher proportion of minority adolescents who were flossing had access to floss as opposed to those who were not flossing (86.8% vs. 69.8%, *p* < 0.001). A significantly higher proportion of minority adolescents who were not flossing did not visit the dentist over the past year as opposed to those who floss (25.2% vs. 14.7%, *p* < 0.001). Among the participants who were not flossing, gender, grade level, instruction in school regarding flossing, and multi-theory model (MTM) of health behavior change constructs were the significant predictors (*p* < 0.001) of initiating and sustaining flossing. The findings of this study will serve as baseline data for developing and evaluating effective evidence-based interventions using the MTM.

## 1. Introduction

Oral health is among the essential foundations of good health and is particularly important for adolescents. However, in this age group, oral diseases, including dental caries and periodontal infections, are prevalent [1]. The prevalence of dental caries in the age group 12–19 years, based on National Health and Nutrition Examination Survey (NHANES) data, was 53.8% [2]. Approximately 18.3% of this age group had untreated caries [3]. Further, there has been no significant change in the dental caries rate among adolescents over the past decades [1]. With the onset of puberty, the prevalence of gingivitis and periodontal diseases also increases [4]. According to a previous study, halitosis or bad breath in adolescents was nearly 17.3% [5]. A significant association between halitosis and a lack of daily flossing was also reported [5]. Notably, poor oral health has been associated with poor growth, learning difficulties, behavioral problems [2], and obesity [6]. Therefore, it is imperative to improve the oral health of adolescents.

Adolescents belong to an important age group, as some preventive self-care behaviors (i.e., tooth brushing and flossing) are formed at this stage [7]. One review study suggested that flossing and toothbrushing may help prevent halitosis, dental caries, and periodontal diseases [5,8]. Other studies found that flossing in this age group is associated with decreasing interproximal caries [9] and proximal caries [10]. However, daily flossing is often neglected by adolescents. A study among English adolescents found that flossing became less frequent with age, from 12 to 16 years, and only 6% of adolescents received advice regarding flossing at dental visits [11]. Another study examined oral hygiene among Greek school students and reported that less than 37% of participants used floss [12]. Basch and colleagues (2019) found that adolescents did not floss daily, especially in the evening. Further, the study found that boys associated flossing with health-related behaviors, while girls associated flossing with cleanliness [13]. The NHANES data from 9056 US adults aged 30 years and above reported that approximately 30% floss daily [14]. However, there are no national surveys available that provide an estimate as to what proportion of US adolescents indeed floss. The Behavioral Risk Factor Surveillance system (BRFSS) has no questions regarding flossing behavior, and they do not even appear in state-specific oral health questions. However, data from other countries show flossing to be generally low among adolescents (e.g., England 8%, Canada 22%, and Norway 27%) [11].

Adolescents from minority groups are particularly susceptible to poor oral hygiene behaviors, including lack of daily flossing. A study with Mexican adolescents from rural and urban areas found that rural adolescents were more susceptible to poor oral hygiene behaviors and dental caries [15]. A study with Mexican American teens found that the primary barriers to flossing include the lack of understanding of the proper flossing technique and the messages encouraging flossing [16]. A study found that Brazilian adolescents’ primary barriers to flossing were laziness, lack of motivation, and problems related to manual dexterity [17]. Another qualitative research study performed among rural and low-income minority adolescents in the US reported a lower level of the perceived threat from dental diseases among this group [18]. In addition, this group needed more information and implementation targeting preventive oral health behaviors [18,19]. Studying and promoting oral health among African American/Black and Latinx/Hispanic adolescents is an area of research to reduce health disparity.

Few behavioral and educational interventions have been implemented to promote flossing among adolescents. A low-fear educational intervention based on locus of control theory found no change in flossing compliance among adolescents compared to a control group [20]. Using a cluster randomized controlled trial, a social cognitive theory-based brief intervention with adolescent girls improved flossing behavior with self-efficacy, planning, and intention as the key constructs [21]. However, the intervention was limited to only one gender and had a relatively short follow-up. There is a need for more interventions to promote flossing behavior among adolescents, particularly from minority groups. Conducting theory-based research to identify evidence-based approaches to promote flossing among African American/Black and Latinx/Hispanic adolescents will help reduce oral health disparities affecting these subgroups.

The multi-theory model (MTM) of health behavior change uses salient constructs from several behavioral and social science theories that have proven their usability in explaining several health behaviors among different target groups [22,23]. The MTM can deliver precise and brief interventions for facilitating behavior changes tested in experimental designs. It showed significant and substantial predictability in explaining other behaviors, for instance, promoting physical activity behavior [24,25,26], fruit and vegetables consumption behavior [27], portion size behavior [28], replacing sugar-sweetened beverages with water [29], replacing binge drinking with responsible drinking [30], increasing mammography [31], promoting HPV vaccination [32], reducing water-pipe smoking [33,34], promoting low salt intake among hypertensives [35], promoting good sleep behavior [36], promoting stress management behaviors [37], and promoting nature contact behavior [38].

**Figure 1:**
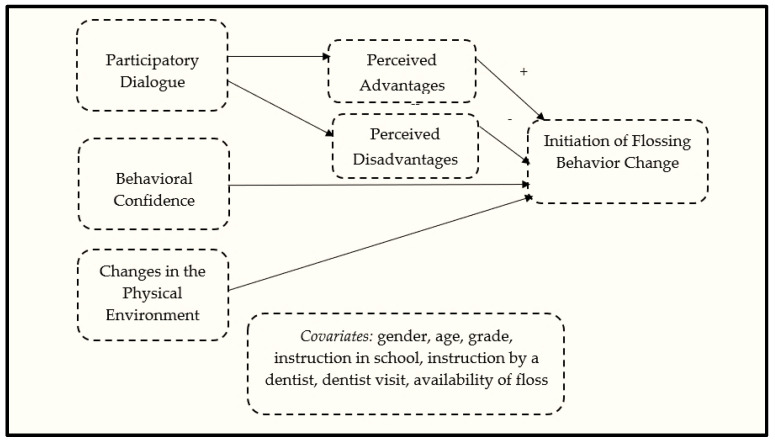
Diagrammatic depiction of the initiation model of the MTM for promoting flossing among African American/Black and Latinx/Hispanic adolescents.

The MTM, unlike previous theories of behavior acquisition, is about behavior change. It conceptualizes the behavior change into two components: (a) initiation and (b) sustenance or maintenance. There are distinct and parsimonious constructs for each component. For initiation, three constructs help with behavior change. The first one is participatory dialogue in which the advantages of the behavior change must be emphasized over the disadvantages. This has been derived from value expectancy theories. The second construct is behavioral confidence, derived from Bandura’s social cognitive theory [39,40], and the construct of perceived behavioral control is from the theory of planned behavior [41]. However, behavioral confidence is a little different than the concept of self-efficacy (behavior-specific confidence emanating from self) and perceived behavioral control (how much a person feels they are in command of enacting a behavior) in several ways: First, behavioral confidence is not just restricted to the self but can come from outside influences too, such as belief in a higher power, confidence in a deity, and confidence in a powerful other. Second, behavioral confidence is not “here and now”, like self-efficacy, but can be futuristic, and one may have the belief to acquire it over time. The third construct for the initiation model is the changes in the physical environment, which refers to the tangible resources necessary for starting the behavioral change. In our study, both the actual availability of floss (used as a covariate) and the construct of “changes in the physical environment”, operationalized as the perceived situational availability or the degree of surety that participants could floss while traveling and that floss had a place in the house and was easily accessible, were used. Figure 1 depicts the MTM-based initiation of flossing in African American/Black and Latinx/Hispanic adolescents.

**Figure 2:**
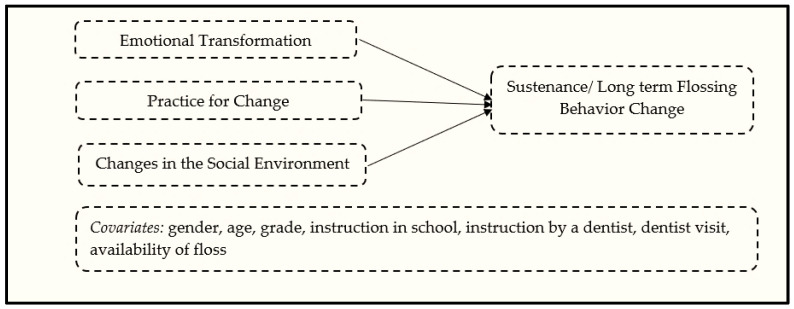
Diagrammatic depiction of the sustenance model of the MTM for promoting flossing among African American/Black and Latinx/Hispanic adolescents.

Likewise, for sustenance or maintenance of behavior changes in the MTM, there are three constructs. The first one is emotional transformation derived from emotional intelligence theory, which requires transforming one’s emotions into goals for making the behavior change. The second construct is practice for change, derived from Freirean praxis [42]. It entails constant thinking about the behavior change or active reflection and reflective action for incorporating it into one’s life. The final construct for the sustenance model is changes in the social environment, which requires social support from family, friends, professionals, and others to continue with the behavior change. These are shown in Figure 2, as regard the flossing behavior of African American/Black and Latinx/Hispanic adolescents. Given the lack of a robust theoretical model to measure flossing behavior, this study served two objectives: first, to develop/validate a survey tool based on the fourth-generation MTM framework and, second, to test the applicability of the MTM in explaining flossing behavior among African American adolescents and Hispanic/Latinx adolescents.

## 2. Methods

### 2.1. Study Design and Eligibility Criteria

This cross-sectional and analytical study was conducted in April 2022 to recruit a sample of African American and Hispanic/Latinx adolescents aged 10–17 years and residing in the US. The representation of the sample mirrored the census distribution by gender and region. Additional inclusion criteria included the ability to understand the English language. The parents signed the informed consent to confirm their children’s participation in the study.

### 2.2. Data Collection Procedure and Sampling

A web-based survey was created using the online survey software Qualtrics (Provo, UT, USA). A survey link was shared with the Qualtrics Market Research Team to field the survey among their panel providers through commercial sampling [43,44]. The potential avenues to distribute the link included but were not limited to the listserv, in-app notification, and special campaigns to meet the quota constraints of the sample. Before fielding the survey link, the Qualtrics team checked all the algorithms or logic set in the survey to ensure the accuracy, and experienced survey fielding experts were involved in the data collection process. Some recommendations to enhance the “look and feel” and a “security check” were made by the Qualtrics team, which were incorporated by the research team. The first few responses were collected from real participants as a part of the “soft launch”. A soft-launch is a term used by the data collection agency that refers to testing an online survey with just a smaller sample size before its broader dissemination in order to find any issues early. This is commonplace for data collection agencies to ensure that the survey works well in real time and that everything is set-up correctly. It also offers the capability to see the response rate and where people are dropping off. The study investigators reviewed the data obtained as a result of the soft launch to identify any discrepancies. Once the data collection was approved for the data quality, a “full launch” of the survey was carried out, and this process continued until the desired quotas were met. As the sample was already constrained by race/ethnicity and age, the remaining quotas by gender (corresponding to the census representation of gender and region) were attained [45].

### 2.3. Ethical Considerations

The university’s IRB approval (UNLV-2021-148, dated 8 April 2022) was obtained before the data collection. Participants from the national Qualtrics pool (an online survey company) were recruited after parental consent and child assent. All data were deidentified, and no personal identifiers were collected. A detailed explanation of the study’s aims, objectives, data collection procedure, risks, and benefits was provided on the informed consent page. Participation was completely voluntary, and participants had complete freedom to quit the study at any point in time. One response per participant was allowed, and it was ensured by the Qualtrics team through security algorithms, such as digital fingerprinting and “prevent multiple responses”. The screening questions related to the participants’ eligibility criteria were posed at the beginning of the survey to prevent any selection bias. Financial compensation was provided to the participants who completed the survey.

### 2.4. Development and Validation of the Survey

**Figure 3:**
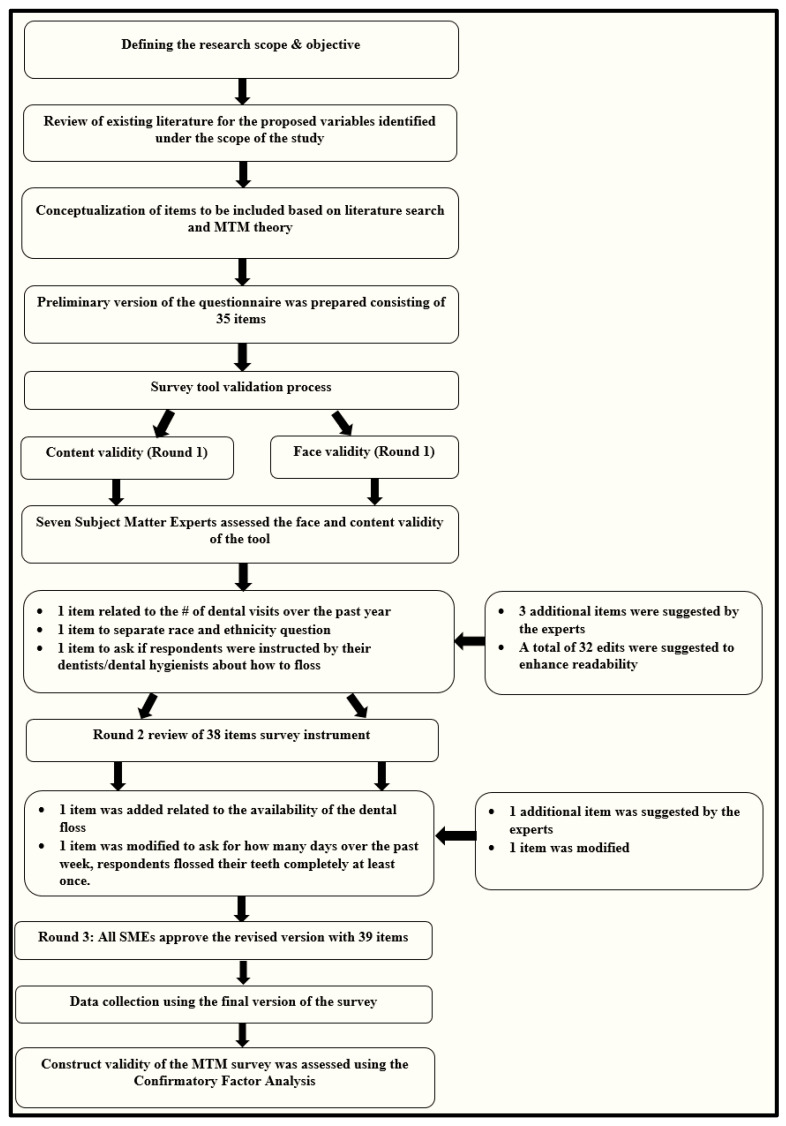
Flow chart depicting the development and validation of the MTM survey tool to measure the change in flossing.

A 39-item questionnaire was developed based on the MTM [22,23] framework, as indicated earlier. For the face and content validity, a total of nine subject matter experts (SMEs) were invited, of which seven responded and helped with the survey’s validation. The survey tool was finalized as a result of three rounds of review by a team of SMEs in the fields of dentistry, instrumentation, behavioral theories (e.g., MTM), and adolescents research (Table 1). The first version of the survey tool consisted of 35 items, which was later modified per the SMEs’ suggestions to include an additional 4 items for a total of 39 items (Figure 3). The instrument finally consisted of demographic information (5 items); flossing behavior (5 items); the perceived advantages of flossing (5 items); the perceived disadvantages of flossing (5 items); behavioral confidence with regard to flossing (5 items); changes in the physical environment related to flossing (3 items); emotional transformation as it pertains to flossing (3 items); practice for change as it relates to flossing behavior (3 items); changes in the social environment related to flossing (3 items); and an item each about the intention of starting (if not a flosser by the question: How likely is it that you will start completely flossing your teeth every day in the next week?) and continuing flossing (by the question: How likely is it that you will completely floss your teeth every day for next one year?”) (total: 39 items). The final instrument had an acceptable Flesch Kincaid Reading Ease score of 71.0 and a Flesh Kincaid reading grade level of 5th grade.

### 2.5. Data Analysis and Sample Justification

The construct validation of the instrument was performed using structural equation modeling (SEM). The model fit indices were calculated. The chi-square (χ^2^), comparative fit index (CFI), root mean square error of approximation (RMSEA), and standardized root mean square residual (SRMR) indices were used to evaluate the overall goodness of fit of the model [46,47]. RMSEA values of ≤0.08 and CFI values greater than 0.95 indicate a good model fit [46]. For the SRMR, values < 0.10 are acceptable, with values < 0.08 as preferable. The SRMR was included because it is the most sensitive to miss-specified factor covariances or latent structures [46]. For establishing the internal consistency, Cronbach’s alpha values were calculated.

Descriptive as well as inferential statistical tests were performed. Bivariate and multivariate tests were utilized to analyze the data. Counts/proportions were used to represent categorical variables, whereas continuous variables are represented as the means and standard deviations. For initiation and sustenance, two models of hierarchical multiple regression analyses were fit to assess the predictive ability of the MTM constructs over the influence of the covariates. Data analyses were performed using IBM SPSS (version 27.0). For all analyses, the alpha was set at 0.05. In calculating the sample size, G*Power for the regression indicated that a minimum of 118 participants was required to achieve a statistical power of 0.80 at an alpha level of 0.05, with a 0.15 (medium) effect size and ten predictors in the equation (three MTM constructs and seven possible covariates in each model) [47,48]. After accounting for a 10% nonresponse bias, the desired sample size was 136 in each group. To perform the structural equation modeling for the construct validation of the subscales, we recruited 260 African American/Black adolescents and 260 Latinx/Hispanic adolescents in this study [49].

## 3. Results

### 3.1. Construct Validation

**Figure 4:**
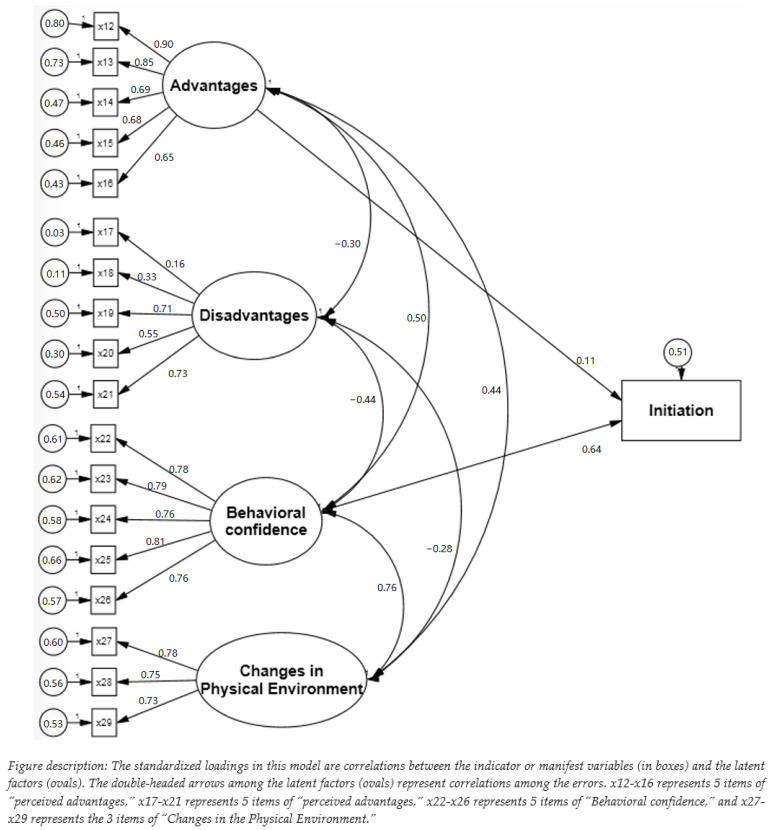
Structural model of initiating flossing.

As shown in the Table 2, the fit indices for the initiation model were in the range of acceptability (e.g., χ^2^[140] = 572.09 (*p* < 0.01), CFI = 0.95, TLI = 0.93, and RMSEA = 0.08). First, we tested the measurement model by examining the factor loadings of four latent variables (i.e., advantages, disadvantages, behavioral confidence, and changes in the physical environment) in the initiation model. The model results indicated that the latent variable of “advantages” had large factor loadings on its five observed variables, which ranged from 0.65 to 0.90. The latent variable of “disadvantages” had small (e.g., β = 0.16) to large (e.g., β = 0.71) factor loadings on its five observed variables. The latent variables of “behavioral confidence” and “changes in the physical environment” also had large effects with loadings, and the βs were all above 0.73 (Figure 4). Based on the measurement model results, the scales for measuring the four constructs of the initiation model had valid measures. Next, we tested the structure model by examining the construct correlations and standardized regression coefficients. We found that “advantages” had a small positive direct effect (β = 0.11, *p* < 0.001) and “behavioral confidence” had a large positive direct effect (β = 0.64, *p* < 0.001) on the initiation of flossing, while the effects of “disadvantages” and “changes in the physical environment” on the initiation were not statistically significant.

**Figure 5:**
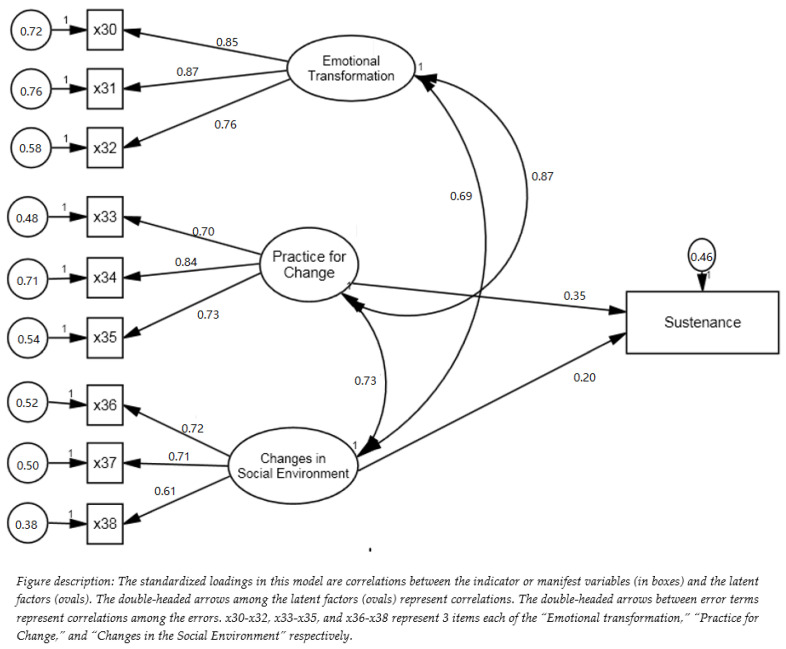
Structural model for sustaining flossing.

For the sustenance model, all the fit indices were consistent with the conventional thresholds for an acceptable fitting model (e.g., χ^2^[30] = 75.50 (*p* < 0.001), CFI = 0.99, TLI= 0.99, and RMSEA = 0.05) (Table 2). The substance model included three latent variables (i.e., emotional transformation, practice for change, and changes in the social environment). The model results indicated that the latent variable of “emotional transformation” had large factor loadings on its three observed variables, which ranged from 0.76 to 0.87. The latent variable of the “practice for change” also had large effects with loadings ranging from 0.70 to 0.84. The third latent variable of the “changes in the social environment” showed medium to large factor loadings, the βs of which ranged from 0.61 to 0.72 (Figure 5). Based on the measurement model results, the sustenance model provided a valid measurement of its three latent constructs. Next, we tested the structure model by examining the construct correlations and standardized regression coefficients for the sustenance model. We found that “practice for change” and “changes in the social environment” both had small direct effects on the sustenance of flossing (β ranging from 0.20 to 0.35). However, the “emotional transformation” did not have a significant effect on the sustenance of flossing.

### 3.2. Intercorrelations and Reliability Diagnostics

We then tested the intercorrelations among the latent constructs and the internal reliability. As shown in Table 3, the results indicated an overall pattern of statistically significant (*p* < 0.05) positive correlations among the latent constructs, except for the correlation between “advantages” and “disadvantages”. We also found the small to medium negative correlations (e.g., *r* coefficient ranging from −0.12 to −0.32) between “disadvantages” and all other latent constructs. In this study, the scale reliability analysis indicated that all scales held sufficient internal consistency (e.g., Cronbach’s alphas ranging from 0.62 to 0.86).

### 3.3. Univariate and Bivariate Statistical Findings

A group of 260 African American/Black and 260 Latinx/Hispanic adolescents (in the ages 10–17 years) from the national Qualtrics pool (an online survey company) were recruited. The proportion of flossing was nearly equally split in the overall sample as well as among African Americans and Latinx/Hispanics (Table 4). The mean age of the sample was 15.82 ± 1.43 years. Over 85% of the sample constituted high school students. Approximately three-quarters of the sample reported having access to floss. Interestingly, over 70% of the sample indicated that they did not have instruction in school regarding flossing (Table 4); however, 83.3% reported being instructed by their dentists. Twenty percent of the sample reported not having a dental visit over the past year (Table 4). As indicated in Table 5, a significantly higher proportion of minority adolescents who were flossing had access to floss as opposed to those who were not flossing (86.8% vs. 69.8%, *p* < 0.001). A significantly higher proportion of minority adolescents who were not flossing did not visit the dentist over the past year as opposed to those who were flossing (25.2% vs. 14.7%, *p* < 0.001) (Table 5).

As shown in Table 6, the mean scores of “behavioral confidence” and “change in the physical environment” were significantly higher among those who were already flossing as opposed to those who were not flossing. On the contrary, the mean score of “perceived disadvantages” was lower among participants who flossed compared to those who did not, although no significant differences were found in the mean scores of the “perceived advantages” among both groups (Table 6).

### 3.4. Hierarchical Multiple Regression

Among the participants who reported flossing, instruction in school regarding flossing, grade level, and all of the MTM subscales, including “emotional transformation”, “practice for change”, and “changes in the social environment”, were significant predictors of sustaining flossing and explained 25.9% of the variance in the flossing among this group (R^2^ = 0.308, F (17, 240) = 6.293, *p* < 0.001; adjusted R^2^ = 0.259) (model 4, Table 7). Upon adding “practice for change” in model 3, the prediction of sustaining flossing improved with a statistically significant increase in the R^2^ of 0.029, F (1, 241) = 9.849, *p* = 0.002. Further, after adding “emotional transformation” (model 2), there was a statistically significant increase in the R^2^ of 0.167, F (1, 242) = 54.141, *p* < 0.001 (Table 7).

Among the participants who were not flossing, the two models of hierarchical regression were fitted with the initiation and sustenance dependent variables, respectively. As shown in the initiation’s final model (Table 8), 33.5% of the variance was explained by the independent variables, including gender, instruction in school regarding flossing, and behavioral confidence (one of the MTM constructs). These variables had more predictive power than other variables (R^2^ = 0.378, F (17, 244) = 8.720, *p* < 0.001; adjusted R^2^ = 0.335). Among the same group, instruction in school regarding flossing, grade level, and all of the MTM subscales, including “emotional transformation”, “practice for change”, and “changes in the social environment”, were significant predictors of sustaining flossing and explained 37.8% of the variance in the flossing among this group (R^2^ = 0.418, F (17, 244) = 10.3222, *p* < 0.001; adjusted R^2^ = 0.378) (model 4, Table 9). Upon adding “practice for change” in model 3, the prediction of sustaining flossing improved with a statistically significant increase in the R^2^ of 0.056, F (1, 245) = 22.715, *p* <0.001. Further, after adding “emotional transformation” (model 2), there was a statistically significant increase in the R^2^ of 0.209, F (1, 246) = 78.001, *p* < 0.001 (Table 9). The two variables in model 1 also improved the prediction with an increase of 0.134 in the R^2^, F (1, 247) = 2.725, *p* = 0.001.

## 4. Discussion

Our study aimed to explain flossing behaviors utilizing the MTM among adolescents from the minority African American and Hispanic/Latinx communities. The study found that 50.4% of the adolescents were not flossing their teeth at least once daily and 21.7% did not have access to floss. As expected, it was also confirmed that the availability of floss made a significantly higher proportion (87%) of minority adolescents floss. As pointed out earlier, there are no national surveys that report flossing rates among adolescents; however, NHANES data among adults demonstrate that 30% of adults do not floss [14]. Our study’s finding of higher rates among minority adolescents points to the need for greater programmatic efforts toward the promotion of flossing in this target group, including making floss available to this group. It is also worth noting that a large majority of the adolescents (71%) received no instruction in school regarding flossing, which again points to the need for greater educational efforts through schools in promoting flossing behavior among minority adolescents. Further, approximately 25% of minority adolescents who did not floss had not visited the dentist over the past year, as opposed to 15% who were flossing. This finding points to the need to make dental care more accessible and affordable to the minority subgroup.

Regarding the MTM constructs, as expected, the mean scores on all constructs were statistically significantly higher for those who were already flossing compared to those who were not. For the minority adolescents who were not flossing, the MTM construct of behavioral confidence along with instruction of flossing in school played an important predictive role, accounting for 37.8% of the variance, which is in the higher range for behavioral studies [22,23]. Behavioral confidence is the ability of the adolescent to have the surety to perform the flossing. Such confidence is futuristic and can arise from self or powerful others, or belief in a higher power or any other such influence. This can be built by exploring and building on sources of confidence for the adolescent, helping them gain mastery over the skill of flossing, teaching flossing techniques in small steps, helping them overcome anxiety or other barriers related to changing the habit of flossing, and other such measures. This finding regarding the role of behavioral confidence is supported by several studies utilizing the MTM with different behaviors [50,51,52]. Further, the inferential finding substantiated the descriptive findings regarding the role that instruction in school settings can play for minority adolescents in changing their flossing behavior. Teachers, school nurses, guest speakers, school health educators, and others in the schools can help minority adolescents develop flossing behavior through concerted messaging.

Among those minority adolescents who were flossing as expected, the intention to sustain flossing was significantly predicted by the three constructs of the MTM, namely, emotional transformation, practice for change, and changes in the social environment along with the grade level and instruction in school and accounted for almost 31% of the variance, which is a higher range for behavioral studies [22,23]. Among those who were not flossing, also, all three constructs of the MTM, namely, emotional transformation, practice for change, and changes in the social environment, were statistically significant predictors along with instruction regarding flossing in school and being African American and accounted for a substantial proportion of variance (42%), which is high for behavioral studies [22,23]. Emotional transformation is about changing the feelings toward the goal of daily flossing and is crucial for maintaining behavior, which was supported by this study and found to be a significant construct in many other studies [26,34,35,37,38]. Adolescents can be taught to recognize their feelings and direct them toward the concrete goals of flossing. Such an approach will strengthen the maintenance of the flossing habit, which has a high relapse rate. Practice for change was found to be significant in this study and has also been an important construct in many other studies with the MTM [26,34,35,37,38]. Thinking about flossing by adolescents and thinking of ways to overcome barriers in the process are a part of the practice of change and are essential for the habit formation of flossing. Educational programs must build on this aspect. Finally, the construct of changes in the social environment was found to be significant in this study and has also been an important construct in many other studies with the MTM [26,34,35,37,38]. It is important for family, peers, and other professionals, including teachers, dental hygienists, dentists, and primary care providers, to underscore the importance of flossing among minority adolescents.

### 4.1. Implications for Practice

The study underscored the need for education in schools regarding flossing for minority adolescents. This task can be built into the curriculum and imparted by the teachers or separately by the school nurses. Additionally, guest lectures by dental hygienists to schools can be arranged. As pointed out earlier, Aguirre-Zero et al. (2016) found that not knowing the proper technique for flossing and a lack of messages regarding flossing were barriers among Mexican children, and a study by Mattos-Silveira et al. (2017) pointed to a lack of motivation and lack of skills [16,17]. Our findings also supported this gap and underscored the importance of behavioral confidence in starting to floss. Further, our study pointed out the need for the availability and accessibility of floss among minority subpopulations. Here, the corporate sector and governmental subsidies can potentially play a role in making floss available to all, irrespective of their being able to afford it. The three constructs of the MTM, namely, emotional transformation, practice for change, and changes in the social environment, will go a long way in building the habit of flossing and must be the cornerstone of all educational interventions. Finally, our study found that visits to dentists also play a potentially important role in facilitating flossing behavior, as shown by a higher proportion of the adolescents flossing in this subgroup. Once again, dentists can provide free visits for those who cannot afford to pay, insurance companies can extend dental visits at low premiums, governmental subsidies can be instituted, etc., to promote access to dental care.

### 4.2. Strengths and Limitations

Our study was the first study to utilize a fourth-generation behavioral model to explain the correlates of flossing among minority adolescents. This study will pave the way for designing robust educational interventions for the promotion of flossing in schools and other settings. However, there were some limitations to our study. The data were collected by self-reports, which have the potential for several biases, such as dishonesty, exaggeration, under-reporting, acquiescence bias, recall bias, and social desirability bias. However, for gauging attitudes, this is the only way to collect data. In this study, intention for flossing was used as a proxy measure of actual flossing. Future studies can utilize experimental designs with interventions to gauge actual behavior change. Further, we did not test for stability reliability in our study. Future studies can conduct the test–retest reliability of the instrument, especially before conducting interventional studies. Finally, the cross-sectional study design did not allow for establishing causality, as both the independent and dependent variables were measured at the same point in time. Future work with interventional experimental studies can overcome this limitation.

## 5. Conclusions and Future Recommendations

Based on this study, it can be concluded that the MTM appears to be a useful framework for explaining the salient correlates of flossing among adolescents from the minority African American and Hispanic/Latinx communities. A large majority of the minority adolescents were not receiving instruction regarding inculcating flossing behavior in schools, which was found to be a strong predictor in our study. This is an identified gap in school health education from this study. The constructs of the MTM can be interwoven effectively in interventions to promote flossing in this target population to be delivered in schools. Additionally, educational interventions must be imparted at dental clinics and other outlets along with system-wide changes to make oral health universally accessible. In addition, there is a dearth of national data on the prevalence of flossing among white adolescents, which could be compared with the data presented in this study. In fact, there is no question related to flossing in the Behavioral Risk Factor Surveillance System (BRFSS). Further additional questions regarding oral health were added in the “state added questions” module but none regarding flossing. This warrants a follow-up study which will allow comparability of the flossing rates among whites and other minority racial groups.

## Figures and Tables

**Table 1:** Details regarding areas of expertise of SMEs involved in the face and content validation of the survey tool.

Area of Expertise	Deidentified Code of SMEs
Adolescents research and instrumentation	Subject matter expert #1
Adolescents research and instrumentation	Subject matter expert #2
MTM and instrumentation	Subject matter expert #3
Adolescents research and MTM	Subject matter expert #4
Adolescents research, instrumentation, behavioral theories	Subject matter expert #5
Adolescents research and public health dentistry	Subject matter expert #6
Adolescents research and general dentistry	Subject matter expert #7

**Table 2:** Values of selected fit statistics for the hypothesized measurement and full structural models.

Hypothesis/Model	Χ^2^	df	RMSEA (90% CI)	CFI	TLI
Initiation model	572.09 **	140	0.08 (0.07–0.08)	0.95	0.93
Sustenance Model	75.50 **	30	0.05 (0.04–0.07)	0.99	0.99

** *p* < 0.01.

**Table 3:** Summary of bivariate correlations, means, standard deviations, and internal consistency estimates for the study variables.

Variables	1	2	3	4	5	6	7
1. Advantages	-						
2. Disadvantages	−0.08	-					
3. Behavioral Confidence	0.36 **	−0.29 **	-				
4. Physical Environment	0.31 **	−0.17 **	0.61 **	-			
5. Emotional Transformation	0.33 **	−0.22 **	0.70 **	0.54 **	-		
6. Practice for Change	0.26 **	−0.32 *	0.61 **	0.54 **	0.69 **	-	
7. Changes in Social Environment	0.24 **	−0.12 **	0.50 **	0.42 **	0.52 **	0.53 **	-
M	14.75	20.88	16.11	10.15	9.97	9.11	9.30
SD	3.48	4.14	5.15	3.22	3.14	3.00	3.04
α	0.85	0.62	0.86	0.75	0.83	0.76	0.66

*n* = 520, * *p* < 0.05; ** *p* < 0.01.

**Table 4:** Characteristics of the minority adolescents who participated in the study (*N* = 520).

Variable	Categories	Descriptive Statistics Overall Sample (*N* = 520)	Hispanic/Latinx 260 (50.0)	African American 260 (50.0)
Gender	Male	246 (47.3)	113 (43.5)	133 (51.2)
	Female	261 (50.2)	135 (51.9)	126 (48.5)
	Other	13 (2.5)	12 (4.6)	1 (0.4)
Age in years (mean ± SD)	-	15.82 ± 1.43	15.8 ± 1.43	15.85 ± 1.44
Region	Midwest	80 (15.4)	35 (13.5)	45 (17.3)
	Northeast	109 (21.0)	56 (21.6)	53 (20.4)
	South	214 (41.2)	78 (30.1)	136 (52.3)
	West	116 (22.3)	90 (34.7)	26 (10.0)
Grade in school	Grade 5	8 (1.5)	4 (1.5)	4 (1.5)
	Middle school (Grade 6–8)	56 (10.8)	26 (10.0)	30 (11.5)
	High school (Grade 9–12)	449 (86.3)	225 (86.5)	224 (86.2)
	Graduated high school	7 (1.3)	5 (1.9)	2 (0.8)
Flossing at least once daily	Yes	258 (49.6)	133 (51.2)	125 (48.1)
	No	262 (50.4)	127 (48.8)	135 (51.9)
Availability of floss	Yes	407 (78.3)	202 (77.7)	205 (78.8)
	No	113 (21.7)	58 (22.3)	55 (21.2)
Instruction in school about flossing	Yes	151 (29.0)	66 (25.4)	85 (32.7)
	No	369 (71.0)	152 (58.5)	140 (53.8)
Instructed by the dentist/dental hygienist about flossing	Yes	433 (83.3)	220 (84.6)	213 (81.9)
	No	87 (16.7)	34 (13.1)	28 (10.8)
Dental visits over the past year	None	104 (20.0)	50 (19.2)	54 (20.8)
	One	136 (26.2)	70 (26.9)	66 (25.4)
	Two	169 (32.5)	78 (30.0)	91 (35.0)
	≥Three	111 (21.3)	62 (23.8)	49 (18.8)

**Table 5:** Comparing characteristics of minority adolescents who floss vs. those who do not floss (*N* = 520).

Variable	Categories	Minority Adolescents Engaged in Flossing (*N* = 520)		*p*-Value
		Yes, 258 (49.6)	No, 262 (50.4)	
Gender	Male	116 (45.0)	130 (49.6)	0.06
	Female	139 (53.9)	122 (46.6)	
	Other	3 (1.2)	10 (3.8)	
Age (mean ± SD)	-	15.72 ± 1.57	15.92 ± 1.27	0.10
Region	Midwest	47 (18.3)	33 (12.6)	0.20
	Northeast	58 (22.6)	51 (19.5)	
	South	97 (37.7)	117 (44.7)	
	West	55 (21.4)	61 (23.3)	
Grade in school	Grade 5	7 (2.7)	1 (0.4)	0.10
	Middle school (Grade 6–8)	31 (12.0)	25 (9.5)	
	High school (Grade 9–12)	217 (84.1)	232 (88.5)	
	Graduated high school	3 (1.2)	4 (1.5)	
Availability of floss	Yes	224 (86.8)	183 (69.8)	**<0.001**
	No	34 (13.2)	79 (30.2)	
Instructed in school about flossing	Yes	78 (30.2)	73 (27.9)	0.70
	No	180 (69.8)	189 (72.1)	
Instructed by the dentist/dental hygienist on how to floss	Yes	223 (86.4)	210 (80.2)	0.06
	No	35 (13.6)	52 (19.8)	
Dental visits over the past year	None	38 (14.7)	66 (25.2)	**<0.001**
	One	52 (20.2)	84 (32.1)	
	Two	98 (38.0)	71 (27.1)	
	≥Three	70 (27.1)	41 (15.6)	

Significant *p*-values are bolded in the table.

**Table 6:** Comparing mean scores of the MTM constructs among minority adolescents who floss vs. who do not (N = 520).

Variables	Possible Range (Minimum, Maximum)	Minority Adolescents Engaged in Flossing (N = 520)		*p*-Value
		Yes, 258 (49.6)	No, 262 (50.4)	-
		M ± SD	M ± SD	-
**Intent of Initiation**	(0, 4)	3.92 ± 1.03	2.88 ± 1.17	**<0.001**
1. Perceived advantages	(0, 20)	20.86 ± 4.22	20.91 ± 4.10	0.80
2. Perceived disadvantages	(0, 20)	14.06 ± 3.62	15.44 ± 3.20	**<0.001**
3. Participatory dialogue	(−20 to +20)	6.79 ± 5.70	5.47 ± 5.483	**0.007**
**4**. Behavioral Confidence	(0, 20)	17.92 ± 4.51	14.32 ± 5.12	**<0.001**
**5**. Changes in the Physical Environment	(0, 12)	10.78 ± 2.92	9.53 ± 3.37	**<0.001**
**Intent of Sustenance**	(0, 4)	3.64 ± 1.17	2.45 ± 1.22	**<0.001**
**6**. Emotional Transformation	(0, 12)	10.91 ± 2.78	9.04 ± 3.18	**<0.001**
**7**. Practice for Change	(0, 12)	9.97 ± 2.75	8.27 ± 2.99	**<0.001**
**8**. Changes in the Social Environment	(0, 12)	9.86 ± 2.84	8.75 ± 3.13	**<0.001**

M = mean; SD = standard deviation. Significant *p*-values are bolded in the table.

**Table 7:** Hierarchical multiple regression to predict the likelihood of sustaining flossing among participants who already initiated flossing (N = 258).

Variable	Model 1		Model 2		Model 3		Model 4	
			Sustenance as a Dependent Variable					
	B	β	B	β	B	β	B	β
Constant	2.808 *	-	1.512	-	1.219	-	0.8767	-
Age	0.069	0.102	0.065	0.097	0.073	0.108	0.078	0.115
Gender: male (Ref: Female)	−0.096	−0.045	−0.141	−0.066	−0.166	−0.078	−0.142	−0.067
Other gender (Ref: Female)	0.127	0.013	0.032	0.003	−0.093	−0.009	−0.165	−0.017
African American (Ref: Hispanic)	−0.036	−0.017	−0.013	−0.006	0.005	0.002	−0.001	−0.001
Grade level: middle school (Ref: Grade 5)	−0.912	−0.280	−0.926 *	−0.284	−0.991 *	−0.304	−0.952 *	−0.292
High school	−0.670	−0.231	−0.643	−0.222	−0.775	−0.267	−0.731	−0.252
Graduated high school	−2.183 *	−0.221	−2.052 *	−0.208	−2.052 *	−0.208	−1.886 *	−0.191
Region: Northeast (Ref: Midwest)	0.254	0.100	0.225	0.089	0.230	0.091	0.198	0.078
South (Ref: Midwest)	0.348	0.159	0.219	0.100	0.235	0.107	0.221	0.101
West (Ref: Midwest)	0.478 *	0.185	0.357	0.138	0.335	0.129	0.329	0.127
Instruction about flossing in school (Ref: No)	0.287	0.125	0.339 *	0.147	0.314 *	0.136	0.279 *	0.121
Instruction about dentists/hygienist (Ref: No)	0.076	0.024	−0.031	−0.010	0.006	0.002	0.007	0.002
Dental visits over the past year: Yes (Ref: No)	−0.086	−0.029	−0.087	−0.029	−0.089	−0.030	−0.147	−0.049
Availability of floss (Ref: No)	0.160	0.051	−0.164	−0.052	−0.168	−0.054	−0.145	−0.046
Emotional transformation	-	-	0.163 **	0.428	0.110 **	0.288	0.089 *	0.233
Practice for change	-	-	-	-	0.086 *	0.224	0.068 *	0.177
Changes in the social environment	-	-	-	-	-	-	0.069 *	0.184
R^2^	0.088	-	0.255	-	0.284	-	0.308	-
F	1.673	-	5.513 **	-	5.973 **	-	6.293 **	-
ΔR^2^	0.088	-	0.167	-	0.029	-	0.024	-
ΔF	1.673	-	54.141 **	-	9.849 *	-	8.459 *	-

* *p*-value < 0.05; ** *p*-value < 0.001. Adjusted R^2^ of model 4 = 0.259.

**Table 8:** Hierarchical multiple regression to predict the likelihood of initiating flossing among participants who were not flossing (N = 262).

Variables	Model 1		Model 2		Model 3		Model 4	
	Initiation as a Dependent Variable							
	B	β	B	β	B	β	B	β
Constant	−2.304	-	−2.122	-	−2.255	-	−2.235	-
Age	0.102	0.110	0.131	0.143	0.128	0.140	0.126	0.137
Gender: male (Ref: Female)	0.315 *	0.135	0.304 *	0.130	0.258 *	0.111	0.259 *	0.111
Other gender (Ref: Female)	−0.282	−0.046	−0.010	−0.002	0.068	0.011	0.072	0.012
African American (Ref: Hispanic)	0.197	0.084	0.220	0.094	−0.015	−0.006	−0.004	−0.002
Grade level: middle school (Ref: Grade 5)	2.561 *	0.644	1.920	0.483	1.167	0.294	1.085	0.273
High school	2.109	0.575	1.380	0.376	0.697	0.190	0.634	0.173
Graduated high school	2.295	0.241	1.374	0.144	0.785	0.082	0.691	0.073
Region: Northeast (Ref: Midwest)	0.292	0.099	0.186	0.063	0.086	0.029	0.041	0.014
South (Ref: Midwest)	0.269	0.114	0.189	0.081	0.206	0.088	0.179	0.076
West (Ref: Midwest)	0.328	0.119	0.208	0.075	0.110	0.040	0.090	0.033
Instruction about flossing in school (Ref: No)	0.461 *	0.177	0.385 *	0.148	0.309 *	0.119	0.301 *	0.115
Instruction about dentists/hygienist (Ref: No)	0.424 *	0.145	0.322	0.110	0.203	0.069	0.210	0.072
Dental visits over the past year: Yes (Ref: No)	0.386 *	0.144	0.405 *	0.151	0.194	0.072	0.180	0.067
Availability of floss (Ref: No)	0.236	0.093	0.141	0.056	−0.011	−0.004	−0.092	−0.036
Participatory dialogue	-	-	0.053 **	0.247	0.021	0.099	0.021	0.098
Behavioral confidence	-	-	-	-	0.111 **	0.487	0.102 **	0.446
Changes in the physical environment	-	-	-	-	-	-	0.031	0.090
R^2^	0.142	-	0.195	-	0.373	-	0.378	-
F	2.911 **	-	3.970 **	-	9.129 **	-	8.720 **	-
ΔR^2^	0.142	-	0.053	-	0.179	-	0.004	-
ΔF	2.911 **	-	16.279 **	-	69.847 **	-	1.735	-

* *p*-value < 0.05; ** *p*-value < 0.001. Adjusted R^2^ of model 4 in initiation = 0.335.

**Table 9:** Hierarchical multiple regression to predict the likelihood of sustaining flossing among participants who were not flossing (N = 262).

Variables	Model 1		Model 2		Model 3		Model 4	
	Sustenance as a Dependent Variable							
	B	β	B	β	B	β	B	β
Constant	−1.595	-	−1.691	-	−1.813	-	−1.805	-
Age	0.087	0.090	0.076	0.080	0.124	0.129	0.104	0.108
Gender: male (Ref: Female)	0.055	0.023	−0.138	−0.057	−0.104	−0.043	−0.133	−0.055
Other gender (Ref: Female)	0.079	0.012	0.454	0.071	0.477	0.075	0.489	0.077
African American (Ref: Hispanic)	0.531 *	0.218	0.358 *	0.147	0.331 *	0.136	0.276 *	0.113
Grade level: middle school (Ref: Grade 5)	1.829	0.441	0.945	0.228	0.214	0.052	0.306	0.074
High school	1.616	0.422	0.887	0.232	−0.023	−0.006	0.130	0.034
Graduated high school	1.901	0.191	1.272	0.128	0.193	0.019	0.373	0.038
Region: Northeast (Ref: Midwest)	0.213	0.069	0.001	0.000	0.015	0.005	0.018	0.006
South (Ref: Midwest)	−0.077	−0.032	−0.229	−0.093	−0.229	−0.093	−0.233	−0.095
West (Ref: Midwest)	0.186	0.065	−0.025	−0.009	−0.003	−0.001	−0.030	−0.010
Instruction about flossing in school (Ref: No)	0.532 *	0.196	0.362 *	0.133	0.332 *	0.122	0.351 *	0.129
Instruction about dentists/hygienist (Ref: No)	0.300	0.098	0.055	0.018	0.010	0.003	−0.043	−0.014
Dental visits over the past year: Yes (Ref: No)	0.362	0.129	0.326 *	0.116	0.283	0.101	0.263	0.094
Availability of floss (Ref: No)	0.027	0.010	−0.128	−0.048	−0.166	−0.063	−0.133	−0.050
Emotional transformation	-	-	0.188 **	0.492	0.100 **	0.261	0.084 *	0.221
Practice for change	-	-	-	-	0.139 **	0.341	0.111 **	0.271
Changes in the social environment	-	-	-	-	-	-	0.071 *	0.181
R^2^	0.134	-	0.342	-	0.398	-	0.418	-
F	2.725 *	-	8.536 **	-	10.129 **	-	10.322 **	-
ΔR^2^	0.134	-	0.209	-	0.056	-	0.020	-
ΔF	2.725 *	-	78.001 **	-	22.715 **	-	8.474 *	-

* *p*-value < 0.05; ** *p*-value < 0.001. Adjusted R^2^ of model 4 in sustenance = 0.378.

## Data Availability

The data presented in this study are available upon request from the corresponding author. The data are not publicly available due to the presence of ethical reasons.
